# S191. INVESTIGATION OF THE PREVALENCE OF COPY NUMBER VARIANT SYNDROMES IN A LARGE SCHIZOPHRENIA COHORT

**DOI:** 10.1093/schbul/sby018.978

**Published:** 2018-04-01

**Authors:** Venuja Sriretnakumar, Clement Zai, James Kennedy, Joyce So

**Affiliations:** 1 University of Toronto; 2 Centre for Addiction and Mental Health; 3 CAMH, University of Toronto; 4 University Health Network, University of Toronto

## Abstract

**Background:**

Many rare genetic syndromes are known to phenotypically manifest with psychiatric symptoms that can be indistinguishable from primary psychiatric disorders. While the majority of ongoing research in psychiatric genetics has been dedicated to the identification and characterization of genes involved in primary psychiatric disorders, there has been a lack of research to determine the extent to which rare genetic variants contribute to the overall psychiatric disease load. In our study, we aim to investigate the prevalence of clinically well-characterized pathogenic copy number variant (CNV) syndromes that are associated with neuropsychiatric phenotypes in a large schizophrenia patient cohort.

**Methods:**

DNA from 348 schizophrenia patients recruited at the Centre for Addiction and Mental Health (CAMH) (Toronto, Canada) was run on the Affymetrix SNP Array 6. 0. CNVs were called using two algorithms (Canary Software and PennCNV) for deletions >200 kb and duplications >500 kb. CNVs called by both algorithms were included in further analysis. All CNVs were individually assessed to determine overlap with known, clinically well-characterized CNV syndromes with the use of the UCSC Genome Browser, DECIPHER GRCh37, and GeneReviews® databases.

**Results:**

A total of 861 deletions and 171 duplications were called on 348 schizophrenia patients. In-depth analysis revealed a total of 16 schizophrenia patients with significant deletions. Microdeletions associated with known syndromes that were identified include: 16p11.2-p12.2 (n=1), 16p13.11 (n=3), 17p11.2 (n=2), 22q11.2 (n=5), 1p36 (n=4), and 5q35.3 (n=1). Analysis for pathogenic microduplications is ongoing.

**Discussion:**

We observed a greater than expected number of syndromic microdeletions amongst the schizophrenia cohort (16/348, 4.6%), particularly CNVs already hypothesized or known to be associated with neurodevelopmental disorders. Screening for these rare genetic disorders could lead to better understanding of the pathophysiology of psychiatric disorders, as well as the prevalence of these syndromic CNVs within various psychiatric population subtypes. Correctly identifying syndromic CNVs within psychiatric populations can improve patient prognosis. Further analyses will be undertaken to define specific genes contained within the implicated CNV regions to better characterize potential genetic effects on the phenotypic presentation of SCZ patients.

